# Supplementary material for: In vitro CSC-derived cardiomyocytes exhibit the typical microRNA-mRNA blueprint of endogenous cardiomyocytes
Source: Commun Biol. 2021 Sep 30;4:1146. doi: 10.1038/s42003-021-02677-y (PMC8484596; doi:10.1038/s42003-021-02677-y)
Supplement: Supplementary file 2 — Supplementary Information [file 42003_2021_2677_MOESM2_ESM.pdf]

In Vitro CSC-derived Cardiomyocytes Exhibit the Typical microRNA-mRNA Blueprint of Endogenous Cardiomyocytes

Mariangela Scalise[1*], Fabiola Marino[1*], Luca Salerno[1], Teresa Mancuso[2], Donato Cappetta[3], Antonella Barone[1], Elvira Immacolata Parrotta[2], Annalaura Torella[3], Domenico Palumbo[4,5], Pierangelo Veltri[2], Antonella De Angelis[3], Liberato Berrino[3], Francesco Rossi[3], Alessandro Weisz[4], Marcello Rota[6], Konrad Urbanek[1], Bernardo Nadal-Ginard[2], Daniele Torella[1†‡], Eleonora Cianflone[2,6†‡]

1. *Department of Experimental and Clinical Medicine, Magna Graecia University, 88100 Catanzaro, Italy;*
2. *Department of Medical and Surgical Sciences, Magna Graecia University, 88100 Catanzaro, Italy*
3. *Department of Experimental Medicine, University of Campania "L. Vanvitelli", 80138 Naples, Italy;*
4. *Department of Medicine, University of Salerno, 84081 Baronissi (Salerno), Italy;*
5. *Clinical Research and Innovation, Clinica Montevergine, 83013 Mercogliano, Italy;*
6. *Department of Physiology, New York Medical College, Valhalla, NY,USA*

* The first two authors equally contribute to this study; †These authors share the seniorship for this study;

‡ **Correspondence** to Daniele Torella, MD, PhD or Eleonora Cianflone, PhD, *Magna Graecia* University, Campus S. Venuta, Viale Europa, 88100, Catanzaro, Italy, Tel: +3909613694185, Fax: +3909613694090, e-mail: dtorella@unicz.it (D.T.) or cianflone@unicz.it (E.C.)

Supplementary information:

- Supplementary Figures 1-5

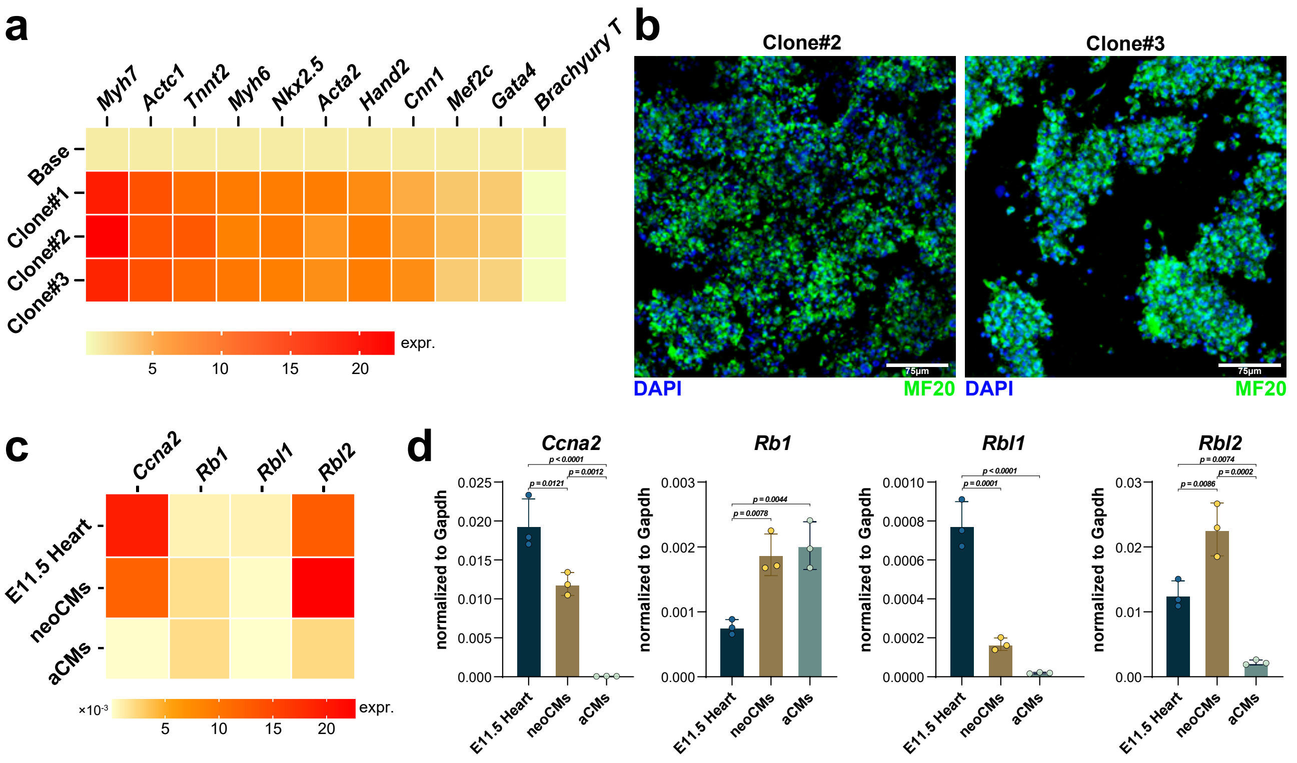

Supplementary Figure 1. Reproducibility of CSC cardiomyocyte (CM) differentiation and expression of the pocket protein family of Rb for cell cycle withdrawal, maturation and terminal differentiation of CMs. (a) Heatmap shows qPCR analysis of main contractile genes in cardiosphere-derived CSCs (*Actc1, Tnnt2, Myh7, Actc1, Myh6, Acta2, Cnn1*) and cardiac transcription factors (*Mef2c, Gata4, Nkx2.5, Hand2* and *Brachyury T*) after myogenic differentiation. **(b)** Representative confocal images of functional cardiomyocytes derived from two different clones of CSCs show a homogenous expression of MF20 (green). Nuclei are stained by DAPI (blue). Scale bar = 75µm. **(c-d)** Heatmap and bar graphs showing RT-PCR data for the expression of the *Ccna2, Rb, Rbl1* and *Rbl2* in E11.5 Heart, neoCMs and aCMs.

The data are expressed as mean ± S.D. of biological triplicates. Each of the biological triplicate was verified by a technical triplicate.

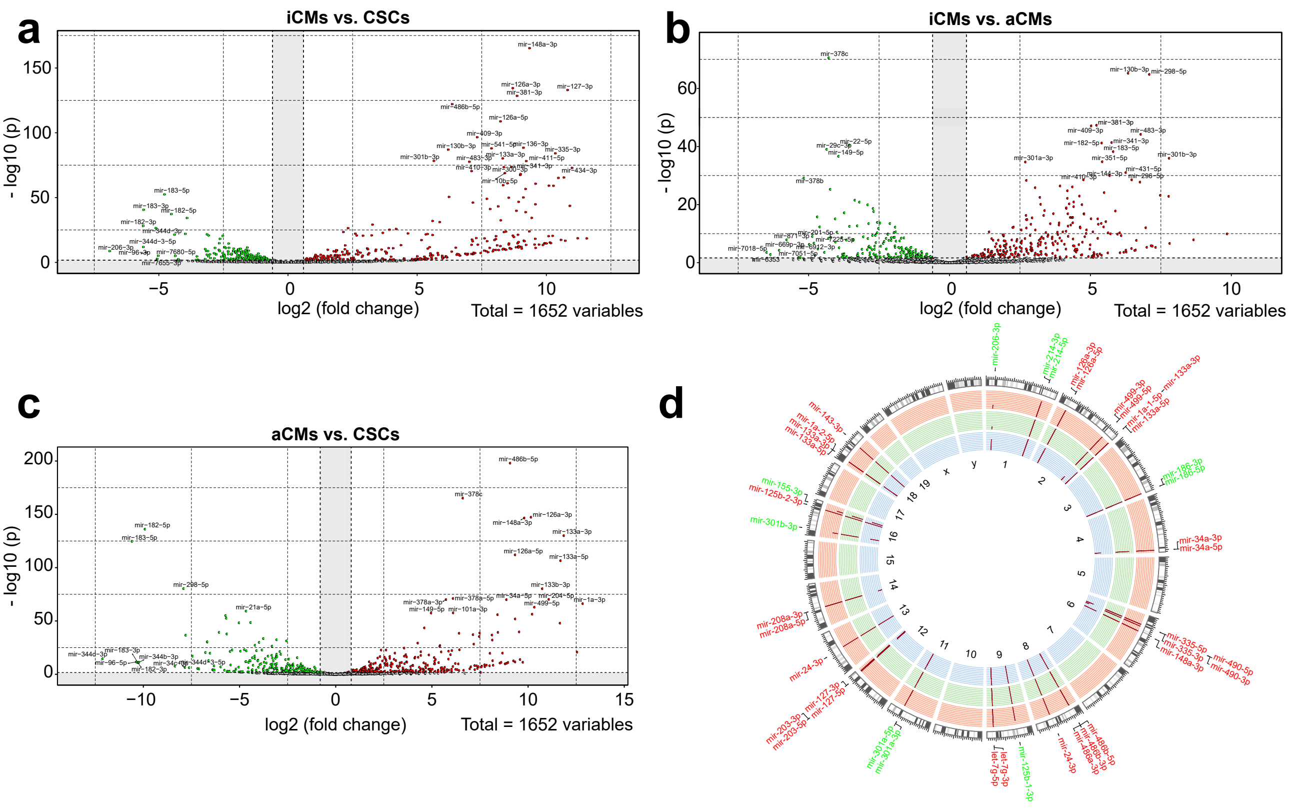

Supplementary Figure 2. miRNASeq Expression of miRNAs. (a-c) Pairwise analysis of miRNAs expression between iCMs vs. CSCs, iCMs vs. aCMs and aCMs vs. CSCs is plotted in the Volcano plots (|FC|1.5 and p ≤ 0.01). Red and green miRNAs show the most significantly up and down-regulated miRNAs, respectively. **(d)** The Circos plot represents the genome localization of the selected myo-miRs.

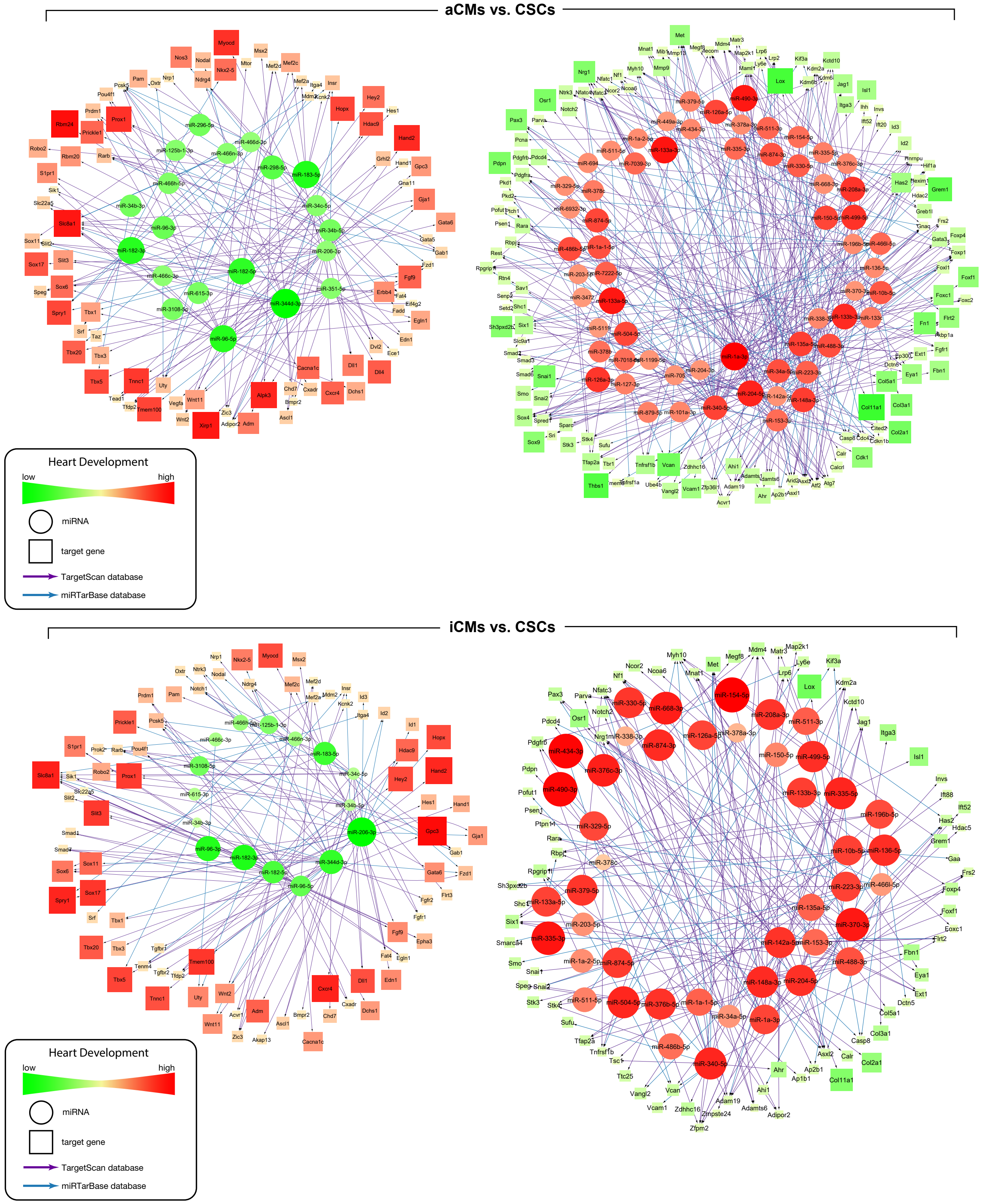

Supplementary Figure 3. miRNA/mRNA networks describing the process of heart development. The networks were built starting from the down-regulated (left) and up-regulated (right) miRNAs in the "aCMs vs. CSCs" (upper panel) and "iCMs vs. CSCs" (bottom panel) comparisons, respectively. The networks were developed inserting each miRNA/mRNA target that was effectively up-regulated for the down-regulated miRNAs and each miRNA/mRNA target that was effectively down-regulated for the up-regulated miRNAs.

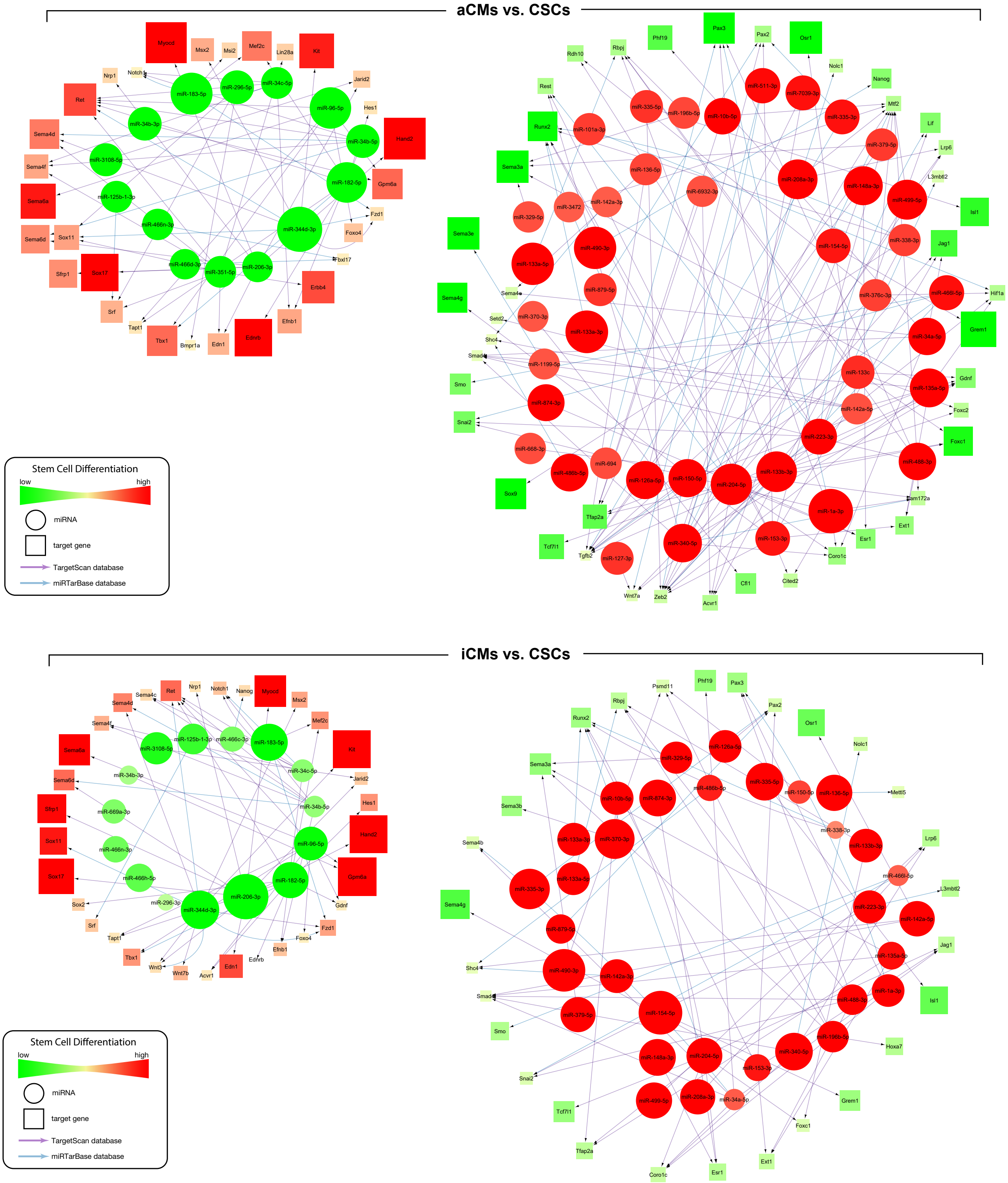

Supplementary Figure 4. miRNA/mRNA networks describing the process of stem cell differentiation. The networks were built starting from the down-regulated (left) and up-regulated (right) miRNAs in the "aCMs vs. CSCs" (upper panel) and "iCMs vs. CSCs" (bottom panel) comparisons, respectively. The networks were developed inserting each miRNA/mRNA target that was effectively up-regulated for the down-regulated miRNAs and each miRNA/mRNA target that was effectively down-regulated for the up-regulated miRNAs.

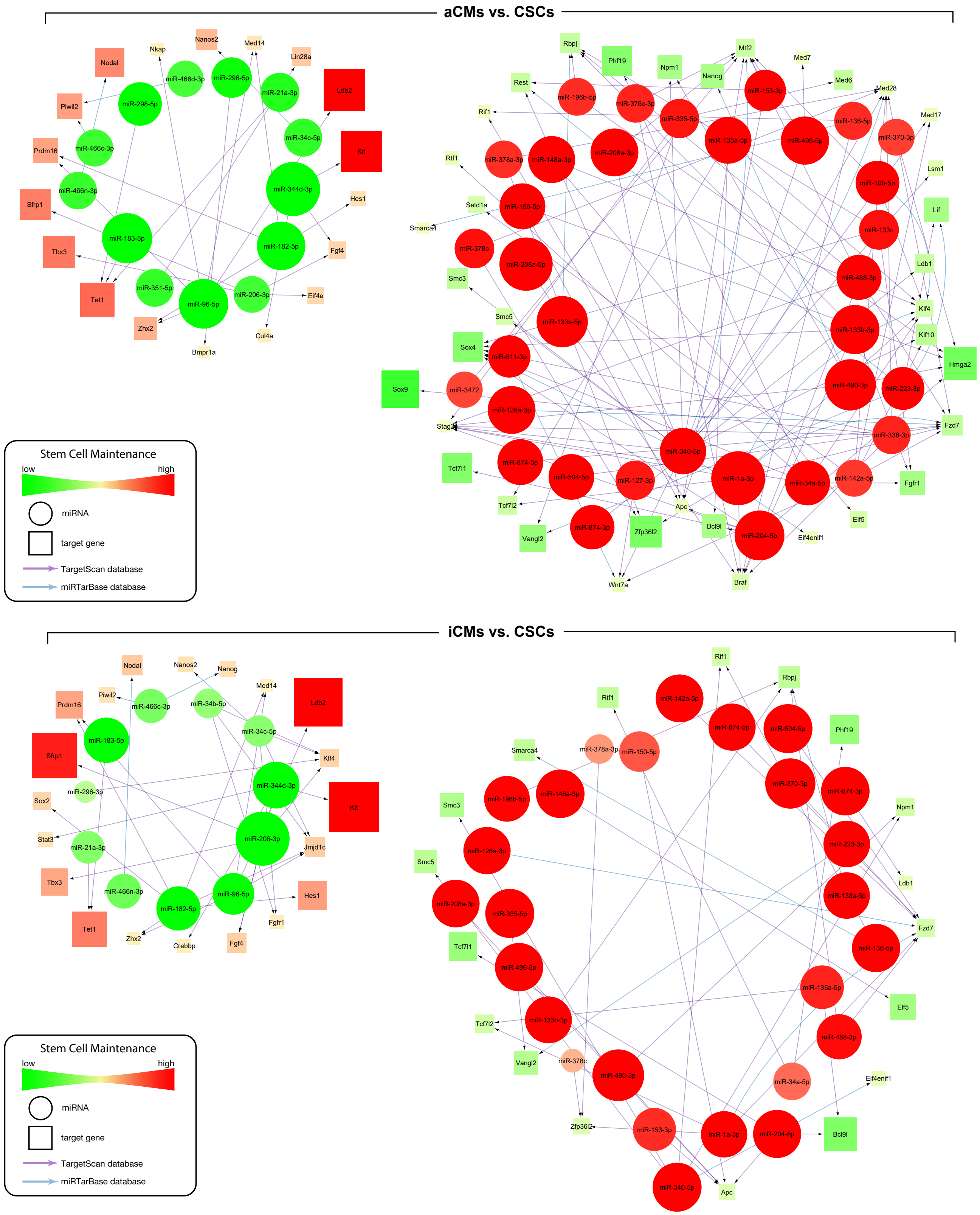

Supplementary Figure 5. miRNA/mRNA networks describing the process of stem cell manteinance. The networks were built starting from the down-regulated (left) and up-regulated (right) miRNAs in the "aCMs vs. CSCs" (upper panel) and "iCMs vs. CSCs" (bottom panel) comparisons, respectively. The networks were developed inserting each miRNA/mRNA target that was effectively up-regulated for the down-regulated miRNAs and each miRNA/mRNA target that was effectively down-regulated for the up-regulated miRNAs.
