# Supplementary material for: In vitro CSC-derived cardiomyocytes exhibit the typical microRNA-mRNA blueprint of endogenous cardiomyocytes
Source: Commun Biol. 2021 Sep 30;4:1146. doi: 10.1038/s42003-021-02677-y (PMC8484596; doi:10.1038/s42003-021-02677-y)
Supplement: Supplementary file 3 — Description of Supplementary Files [file 42003_2021_2677_MOESM3_ESM.pdf]

Description of Additional Supplementary Files

File name: Supplementary Movie 1
Description: High magnification of beating CSC-derived Cardiosphere. This video shows a beating CSC-derived Cardiospheres at high magnification.

File name: Supplementary Data 1
Description: List of selected 176 Cardiomyo-genes

File name: Supplementary Data 2 – 4
Description: List of miRNAs grouped in Cluster 1, Cluster 2 and Cluster 3, respectively in Figure 4b.

File name: Supplementary Data 5.
Description: List of differentially expressed miRNAs in the iCMs vs. CSCs comparison.

File name: Supplementary Data 6
Description: List of differentially expressed miRNAs in the iCMs vs. aCMs comparison.

File name: Supplementary Data 7
Description: miRNA/mRNA interactions between down-regulated (green) miRNAs/up-regulated (red) targets and upregulated (red) miRNAs/downregulated (green) targets in the "aCMs vs. CSCs" comparison involved in the processes of cardiomyocyte differentiation.

File name: Supplementary Data 8
Description: miRNA/mRNA interactions between down-regulated (green) miRNAs/up-regulated (red) targets and upregulated (red) miRNAs/downregulated (green) targets in the "iCMs vs. CSCs" comparison involved in the processes of cardiomyocyte differentiation.

File name: Supplementary Data 9
Description: miRNA/mRNA interactions between down-regulated (green) miRNAs/up-regulated (red) targets and upregulated (red) miRNAs/downregulated (green) targets in the "aCMs vs. CSCs" comparison involved in the processes of cell cycle.

File name: Supplementary Data 10
Description: miRNA/mRNA interactions between down-regulated (green) miRNAs/up-regulated (red) targets and upregulated (red) miRNAs/downregulated (green) targets in the "iCMs vs. CSCs" comparison involved in the processes of cell cycle.

File name: Supplementary Data 11
Description: Source data for the graphs and charts in main and supplementary figures.
