# Supplementary material for: In vitro CSC-derived cardiomyocytes exhibit the typical microRNA-mRNA blueprint of endogenous cardiomyocytes
Source: Commun Biol. 2021 Sep 30;4:1146. doi: 10.1038/s42003-021-02677-y (PMC8484596; doi:10.1038/s42003-021-02677-y)
Supplement: Supplementary file 14 — Reporting Summary [file 42003_2021_2677_MOESM14_ESM.pdf]

nature research

Corresponding author(s): Daniele Torella and Eleonora Cianflone

Last updated by author(s): Aug 31, 2021

Reporting Summary

Nature Research wishes to improve the reproducibility of the work that we publish. This form provides structure for consistency and transparency in reporting. For further information on Nature Research policies, see our Editorial Policies and the Editorial Policy Checklist.

Statistics

For all statistical analyses, confirm that the following items are present in the figure legend, table legend, main text, or Methods section.

n/a	Confirmed	
☐	☒	The exact sample size (*n*) for each experimental group/condition, given as a discrete number and unit of measurement
☐	☒	A statement on whether measurements were taken from distinct samples or whether the same sample was measured repeatedly
☐	☒	The statistical test(s) used AND whether they are one- or two-sided<br>*Only common tests should be described solely by name; describe more complex techniques in the Methods section.*
☒	☐	A description of all covariates tested
☐	☒	A description of any assumptions or corrections, such as tests of normality and adjustment for multiple comparisons
☐	☒	A full description of the statistical parameters including central tendency (e.g. means) or other basic estimates (e.g. regression coefficient) AND variation (e.g. standard deviation) or associated estimates of uncertainty (e.g. confidence intervals)
☐	☒	For null hypothesis testing, the test statistic (e.g. *F*, *t*, *r*) with confidence intervals, effect sizes, degrees of freedom and *P* value noted<br>*Give P values as exact values whenever suitable.*
☒	☐	For Bayesian analysis, information on the choice of priors and Markov chain Monte Carlo settings
☒	☐	For hierarchical and complex designs, identification of the appropriate level for tests and full reporting of outcomes
☒	☐	Estimates of effect sizes (e.g. Cohen's *d*, Pearson's *r*), indicating how they were calculated

Our web collection on statistics for biologists contains articles on many of the points above.

Software and code

Policy information about availability of computer code

Data collection	No custom code/software was used in collection of this data.
Data analysis	Statistical analysis was performed using commercially available Graphpad Prism.

For manuscripts utilizing custom algorithms or software that are central to the research but not yet described in published literature, software must be made available to editors and reviewers. We strongly encourage code deposition in a community repository (e.g. GitHub). See the Nature Research guidelines for submitting code & software for further information.

Data

Policy information about availability of data

All manuscripts must include a data availability statement. This statement should provide the following information, where applicable:

- Accession codes, unique identifiers, or web links for publicly available datasets
- A list of figures that have associated raw data
- A description of any restrictions on data availability

The source data for graphs can be obtained from the corresponding author upon reasonable request. RNA-Seq data that support the findings of this study have been deposited in GEO with the accession code "GSE161081".

Field-specific reporting

Please select the one below that is the best fit for your research. If you are not sure, read the appropriate sections before making your selection.

☒ Life sciences ☐ Behavioural & social sciences ☐ Ecological, evolutionary & environmental sciences

For a reference copy of the document with all sections, see nature.com/documents/nr-reporting-summary-flat.pdf

Life sciences study design

All studies must disclose on these points even when the disclosure is negative.

Sample size	Sample size has not been formally predetermined but was based on previous studies.
Data exclusions	No data were excluded for the data shown.
Replication	Technical triplicates of each biological replicate were performed.
Randomization	Randomization is not applicable for this type of in-vitro study.
Blinding	Blinding is not applicable.

Reporting for specific materials, systems and methods

We require information from authors about some types of materials, experimental systems and methods used in many studies. Here, indicate whether each material, system or method listed is relevant to your study. If you are not sure if a list item applies to your research, read the appropriate section before selecting a response.

Materials & experimental systems

n/a	Involved in the study	
☐	☒	Antibodies
☒	☐	Eukaryotic cell lines
☒	☐	Palaeontology and archaeology
☐	☒	Animals and other organisms
☒	☐	Human research participants
☒	☐	Clinical data
☒	☐	Dual use research of concern

Methods

n/a	Involved in the study	
☒	☐	ChIP-seq
☒	☐	Flow cytometry
☒	☐	MRI-based neuroimaging

Antibodies

Antibodies used	Primary rabbit anti-cardiac troponin I (1:100 dilution, Abcam) and mouse anti-MF20 (1:50 diluition, DSHB). Secondary anti-rabbit-488 and anti-mouse-594 secondary antibody (1:100 dilution, Jackson Immunoresearch).
Validation	Antibody validation provided by manufacturers is available from Abcam (https://www.abcam.com/), DSHB (https://dshb.biology.uiowa.edu/) and Jackson Immunoresearch (https://www.jacksonimmuno.com/). Each antibody was standardly tested.

Animals and other organisms

Policy information about studies involving animals; ARRIVE guidelines recommended for reporting animal research

Laboratory animals	Fetal (from n.3 pregnant mice at E11.5 day), neonatal (from n.3 littermates with 9-10 puppies each at 2 post-natal days) and adult (n.6, 12-weeks-old) C57BL/6J mice were employed in this study.
Wild animals	Not applicable.
Field-collected samples	Not applicable.
Ethics oversight	All animal experimental procedures were approved by Magna Graecia Institutional Review Boards on Animal Use and Welfare and performed according to the 'Guide for the Care and and Use of Laboratory Animals' from directive 2010/63/EU of the European Parliament.

Note that full information on the approval of the study protocol must also be provided in the manuscript.
